# Conserved Translatome Remodeling in Nematode Species Executing a Shared Developmental Transition

**DOI:** 10.1371/journal.pgen.1003739

**Published:** 2013-10-03

**Authors:** Michael Stadler, Andrew Fire

**Affiliations:** 1 Department of Genetics, Stanford University, Stanford, California, United States of America; 2 Department of Pathology, Stanford University, Stanford, California, United States of America; The University of North Carolina at Chapel Hill, United States of America

## Abstract

Nematodes of the genus *Caenorhabditis* enter a developmental diapause state after hatching in the absence of food. To better understand the relative contributions of distinct regulatory modalities to gene expression changes associated with this developmental transition, we characterized genome-wide changes in mRNA abundance and translational efficiency associated with L1 diapause exit in four species using ribosome profiling and mRNA-seq. We found a strong tendency for translational regulation and mRNA abundance processes to act synergistically, together effecting a dramatic remodeling of the gene expression program. While gene-specific differences were observed between species, overall translational dynamics were broadly and functionally conserved. A striking, conserved feature of the response was strong translational suppression of ribosomal protein production during L1 diapause, followed by activation upon resumed development. On a global scale, ribosome footprint abundance changes showed greater similarity between species than changes in mRNA abundance, illustrating a substantial and genome-wide contribution of translational regulation to evolutionary maintenance of stable gene expression.

## Author Summary

Working with a set of four related animal species, we have studied a conserved developmental and metabolic transition at the level of protein production and regulation of RNA levels. Strikingly, regulatory effects at the level of RNA accumulation and protein synthesis act together to achieve the observed metabolic shift. In addition to a general conservation of the underlying basis for the regulation of individual genes, alterations of these two processes—mRNA production and protein synthesis—can compensate for one another during evolution to maintain stable amounts of functional gene products. A salient feature of the observed regulation was the storage of idle mRNAs encoding key members of the protein synthesis machinery during metabolic arrest (diapause). Maintenance of this pool facilitates re-activation upon feeding, with the rapid regeneration of protein synthesis capacity an early and critical function during adaptation to a major metabolic shift.

## Introduction

Animals of diverse genera react to unfavorable growth conditions by entering developmentally arrested states known as diapause [1], [2]. Nematodes of the genus *Caenorhabditis* can enter and exit diapause at several developmental time points, allowing populations to reproduce through boom and bust cycles of nutrient availability. At least four specific programs of developmental arrest and resumption have been identified, each accompanied by unique morphological and gene-regulatory responses [3]–[6]. In newly-hatched *Caenorhabditids*, entry and exit from L1 diapause can be controlled in large and synchronous populations by depriving or providing food. *C. elegans* L1 diapause responses have been well characterized at the level of mRNA biogenesis, with developmental state changes associated with substantial transcriptional changes, the accumulation of RNA polymerase at gene promoters, and alternative splicing [7], [8].

**Figure 1:**
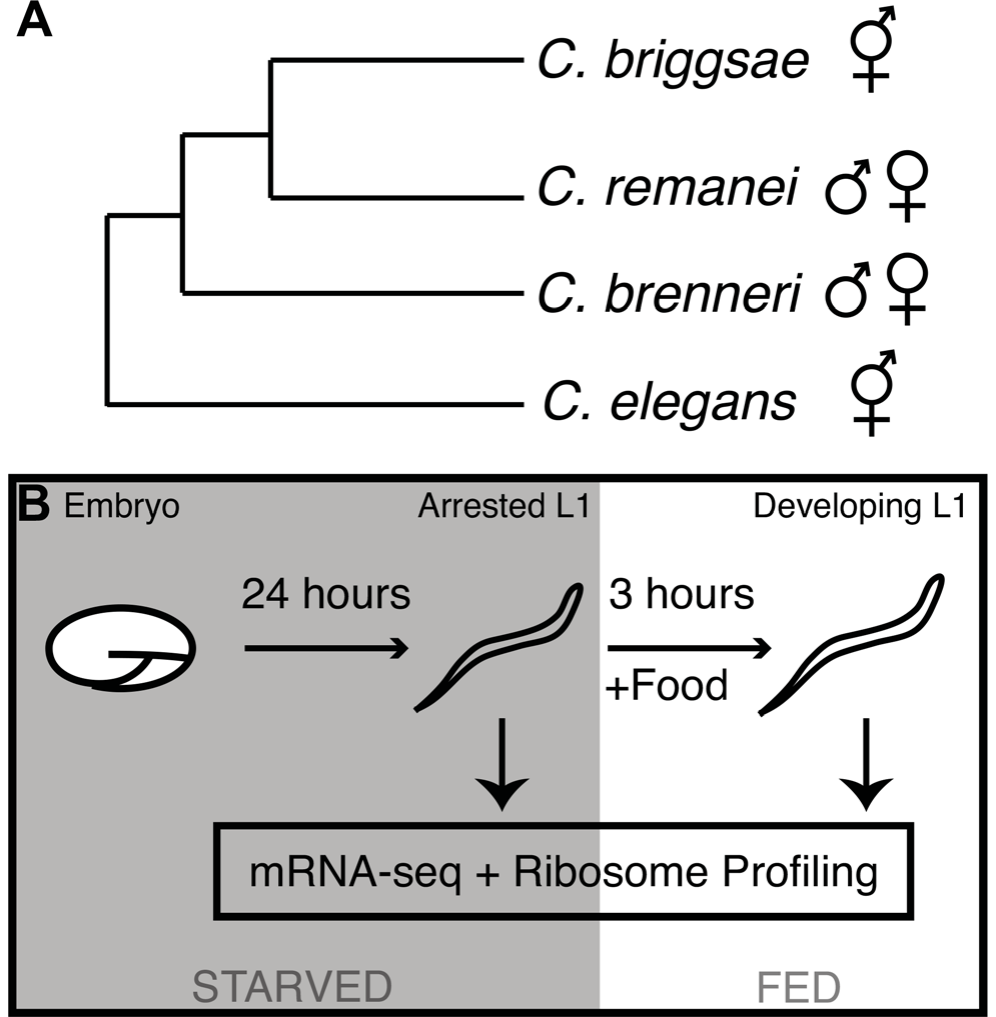
Phylogeny of the *elegans* supergroup and experimental overview. (A) Phylogenetic relationships of the four *Caenorhabditis* species used in this work, according to Kiontke *et al.* [33]. (B) Graphical overview of experimental procedure.

Translational regulation is also expected to contribute significantly to major developmental transitions. We selected four nematode species for investigation of the translation and mRNA-level gene regulatory program associated with L1 diapause exit: two hermaphroditic species, *Caenorhabditis elegans* and *C. briggsae*, and two gonochoristic (male/female) species, *C. remanei* and *C. brenneri* (**Fig. 1**A). These four species exhibit highly similar morphologies and developmental timing despite significant genomic sequence divergence [9]. For each species, we applied mRNA-seq and ribosome profiling [10] to populations of arrested L1 diapause larvae and to populations harvested three hours after the first food encounter that signals the animals to exit diapause and commence development (**Fig. 1**B; sequencing summary statistics: Table S1). Samples from the two conditions are denoted “diapause” or “developing” throughout. All sequence reads and processed count data are available from the Gene Expression Omnibus (http://www.ncbi.nlm.nih.gov/geo) via accession number GSE48140.

Our data provide gene-by-gene measurements at two levels of expression: (i) mRNA-seq data measures the relative steady-state abundances of mRNAs in the transcriptome (abundances are products of mRNA biogenesis and decay), (ii) ribosome profiling allows the counting of ribosome-protected fragments (RPFs) derived from each mRNA, with each fragment corresponding to one active ribosome and thus to an instance of peptide synthesis [10]. For brevity, we use “translatome” [11] to describe the population of mRNA fragments undergoing translation at a given time point, with the relative abundances captured by RPF counts. We emphasize that the abundance of RPFs reflects the input of two biological parameters: steady-state mRNA abundance and ribosome binding (translation efficiency). As diapause entails a substantial decrease in overall translational activity [12], it is additionally important to note that all measurements we infer from the data are relative. Changes in RPF levels between L1 diapause and developing states thus represent relative changes in the commitment of available translational resources between the two conditions.

## Results/Discussion

**Figure 2:**
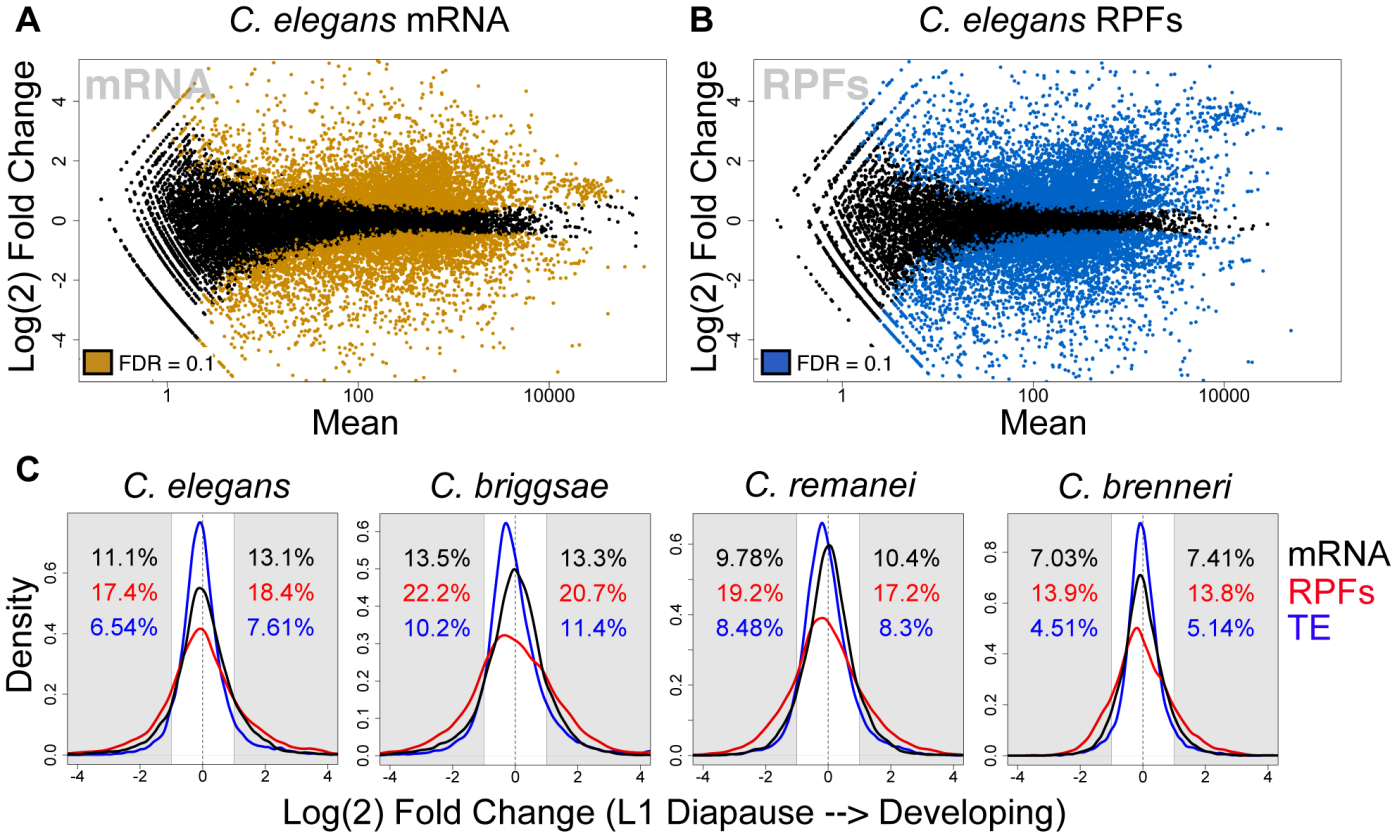
The transcriptome and translatome are substantially remodeled upon exiting diapause. (A–B) Expression mean versus log(2) fold change (MA) plots for *C. elegans* (A) mRNA and (B) RPF abundance changes resulting from feeding-induced diapause release. Colored points represent transcripts determined to be differentially expressed by a negative binomial test (fdr = 0.1) [28]. (C) Distributions of fold changes in mRNA (black) and RPF (red) abundances and translation efficiency (TE: the ratio of RPF:mRNA abundance; in blue). Shaded regions: >2-fold change; black (mRNA), red (RPF), or blue (TE) text indicates percentage of all transcripts showing >2-fold change in the indicated direction. We include the translation efficiency metric for reference, noting that this measure is dependent on the ratio of RPF to mRNA values and is thus not an independent metric of the other two values shown. Controls demonstrating that observed differences are not due to differences in variance of the data types are shown in Fig. S7.

As a starting point for our analysis, we sought to examine the general character of the gene expression changes associated with the transition from L1 diapause to development. mRNA-seq measurements indicated that diapause exit triggered substantial remodeling of transcriptome composition in the four species, similar to the response previously described in *C. elegans* [7], with thousands of differentially expressed transcripts and expression changes spanning three orders of magnitude (**Fig. 2**A, Fig. S1). As expected, transcriptome changes (changes in steady-state mRNA levels) were accompanied by dramatic shifts in the composition of the translatomes (**Fig. 2**B, Fig. S1). The combination of transcriptome and translatome data from common samples allowed us to compare of the relative magnitude of the two levels of response. **Fig. 2**C shows a comparison of the frequency distributions of mRNA and RPF abundance changes for the four species. In each case, we observed a significantly broader distribution for RPF changes, consistent with a regulatory response in which changes in translation efficiencies and mRNA levels taken together constitute a larger magnitude overall response than that seen at the level of mRNA abundance alone (**Fig. 2**C, p<2e-16 for all comparisons). Overall, between the four species we found that ∼15–30% of well-expressed transcripts showed a >2-fold change in relative mRNA abundance, and ∼30–45% of transcripts changed >2-fold in relative RPF abundance.

**Figure 3:**
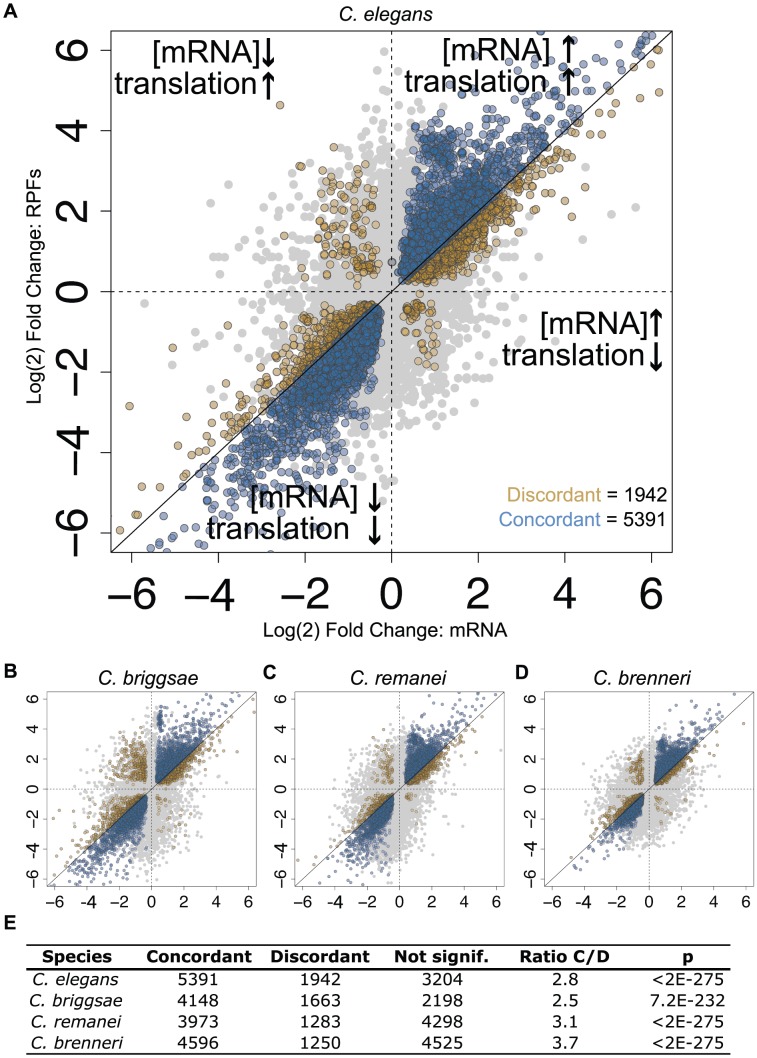
Changes in translation efficiency and mRNA abundance tend to act cooperatively. (A) Log(2) fold-changes for mRNA abundance are plotted against RPF fold-changes for *C. elegans*, with regions inferred to represent concordant and discordant changes in translation efficiency and mRNA abundance indicated. Transcripts called as significantly differentially-expressed in both RPF and mRNA-seq datasets (**Fig. 2**) are shown in color; blue: concordant changes, gold: discordant changes. (B–D) Equivalent plots for the three additional species. (E) Counts of transcripts subject to concordant and discordant changes in the four species as well as the ratio (concordant:discordant) and significance. P-values: binomial test with the null hypothesis that concordant and discordant changes are equally likely (P_concordant_ = P_discordant_ = 0.5). Strongly discordant *C. elegans* transcripts are listed in Table S6.

The transcriptome-wide tendency for RPF level changes to exceed mRNA changes could in principle have resulted from (i) translational changes that act synergistically with and amplify mRNA abundance changes, or (ii) translational changes of large magnitude whose directions are somewhat or predominantly independent of the direction of mRNA abundance changes. While the first scenario may seem most likely from a cellular energetic perspective, genome-wide studies in a variety of systems have revealed varying degrees of coordination (and lack of coordination) of transcriptome and translational responses to a range of stimuli [11], [13]–[15]. To determine which of these scenarios best match our data, we compared changes in RPF level to changes in mRNA level on a gene-by-gene basis. As RPF levels represent the combined input of processes affecting mRNA abundance (e.g., transcription, decay) and translational regulation, we reasoned that, for a given transcript, a change in RPF level in the same direction and of a greater magnitude than the change in mRNA level represents a case in which translation and mRNA abundance processes act in concert (a “concordant” change; equivalent to “homodirectional” in [15]). Conversely, if the change in RPF level is of lesser magnitude or in the opposite direction to the change in mRNA level, this represents a situation in which translational regulation is acting in opposition to mRNA abundance processes (a “discordant” change; **Fig. 3**A). Comparing transcript-wise changes in mRNA and RPF levels from our data, we found that concordant changes were overwhelmingly favored over discordant changes in the four species by a ratio of 2.8–3.7∶1 (**Fig. 3**B–E). Thus during the feeding-induced transition from L1 diapause to active development, these four species apparently utilize a shared regulatory logic: an amplified global gene expression response produced by synergistic changes in mRNA abundance and translational control.

**Figure 4:**
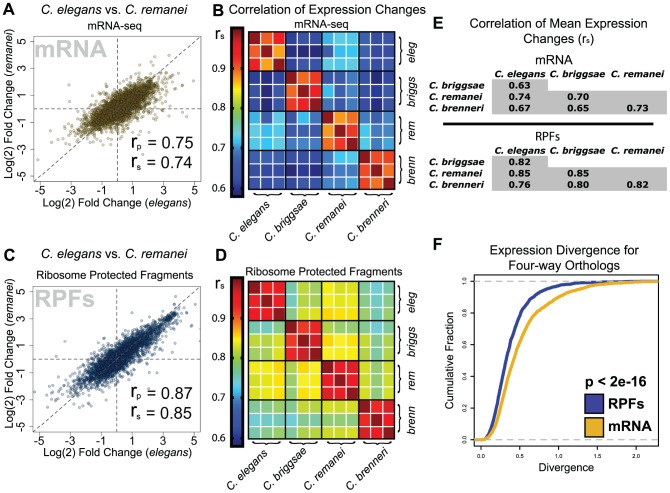
Translatome changes are more similar between species than transcriptome changes. (A) Comparison of mRNA fold changes for orthologous transcripts in *C. elegans* and *C. remanei*. r_p_ = Pearson's correlation coefficient, r_s_ = Spearman's rho. (B) Heatmap of rank correlations of mRNA fold-changes between individual biological replicates for four species. (C–D) Identical figures to A and B using RPF abundance data. (E) Tables of correlation coefficients for species comparisons of mean mRNA and RPF fold changes (mean from three biological replicates; Spearman's rho shown, Pearson coefficients shown in Table S7). (F) Cumulative distributions of expression divergence for mRNA and RPF levels within four-way ortholog groups; p-value: Kolmogorov-Smirnov test.

We next asked to what degree expression changes were similar between species at the gene level. Studies of gene expression conservation have largely focused on mRNA levels [16]–[20], though several groups have reported superior conservation of orthologous protein abundances, implying an important role for translational or post-translational mechanisms in maintaining optimal gene expression during evolution [21], [22]. We began by comparing feeding-induced changes in mRNA abundance for ortholog pairs identified between the four species. mRNA abundance changes for well-expressed transcripts correlated strongly in all pair-wise species comparisons (**Fig. 4**A, Fig. S2). Observed correlation coefficients were highly significant and ranged from 0.63 to 0.74 (Spearman's rho, **Fig. 4**B, E). We next examined the between-species correlation of changes in RPF levels. For each pair-wise species comparison, we observed significantly stronger correlations for changes in RPF abundance than for changes in mRNA abundance, with correlation coefficients in the range of 0.76 to 0.85 (**Fig. 4**C–E, Fig. S2). To complement pair-wise correlations, we examined expression changes within ortholog groups for which an ortholog could be assigned in each of the four species (the four genes together constituting a “four-way” ortholog group). We found that overall expression divergence within four-way ortholog groups was significantly greater for mRNA abundance changes than for RPF changes (**Fig. 4**F, p<2e-16), and that this difference disappeared after randomly shuffling ortholog groupings (Fig. S3). Together, these results demonstrate that, for the L1 diapause program in these nematode species, comparisons accounting for translational regulation reveal a greater level of overall gene expression conservation than is observed at the level of mRNA abundance alone. A significant resulting inference is that alterations in translational control and processes affecting mRNA abundance can compensate for one another during evolution to achieve stable protein expression.

**Figure 5:**
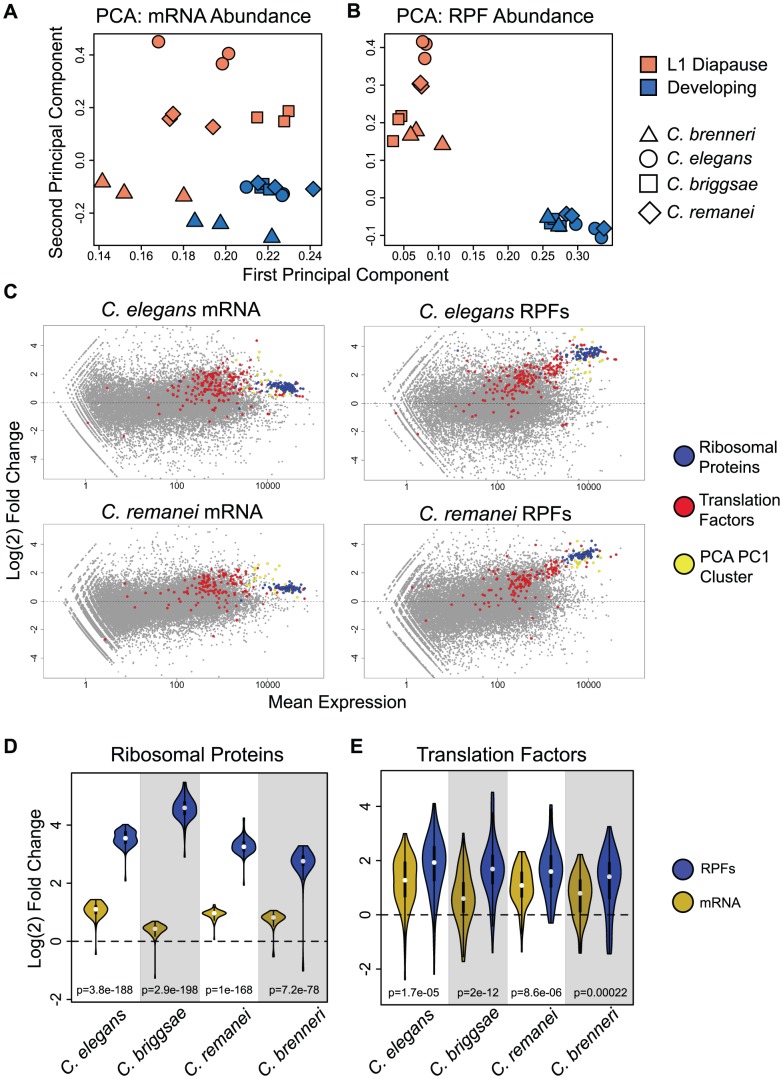
Ribosomal proteins and components of the translation apparatus are potently and coordinately up-regulated at the translation and mRNA level. (A–B) PCA was performed separately on (A) mRNA and (B) RPF abundance datasets consisting of normalized counts for four-way orthologs in individual samples (one dataset for each replicate and condition). Factor loadings on the first two principal components are plotted for each sample. (C) MA plots of mRNA and RPF expression overlaid with transcripts corresponding to non-ribosomal translation factors (red), ribosomal protein genes (blue), and non translation-related genes highly-weighted on the first principal component (yellow). Data is shown for *C. elegans* and *C. remanei*; *C. briggsae* and *C. brenneri* show highly similar patterns (Fig. S5). (D–E) Violin plots [31] of fold changes for (D) ribosomal protein genes and (E) non-ribosomal translation factors in mRNA-seq (gold) and RPF (blue) data. White dots indicate medians, box edges represent the interquartile range, and the colored region and curve shows the probability density function. Significance is derived from two-tailed t-tests.

The substantial observed conservation underscored the functional significance of expression changes during L1 diapause exit. We therefore sought to compare general properties of gene expression in the arrested and developing states, and specifically to identify features that distinguished the two states. Principal components analysis (PCA) is a technique that takes data featuring many variables, such as gene expression data, and extracts a series of linear combinations of the individual variables (called a “principal component”) that explains a substantial fraction of the variance between samples, with each successive component explaining less variance than the previous component. We applied PCA separately to ortholog abundance data from mRNA-seq and RPF datasets, including all arrested and developing samples from the four species in the analysis. For mRNA-seq data, the first two principal components provide poor separation of samples by condition or species (**Fig. 5**A). For RPF data, we observe a clear separation of diapause samples from developing samples on both of the first two principal components (**Fig. 5**B, Fig. S4). The clean separation achieved with RPF data suggests that translatomes of animals from different species in the same nutritional/developmental state are more similar than translatomes from animals of the same species in opposite states.

For RPF data, the first principle component explains a majority of the between-sample variance (86.4%), and higher values on this component are associated with developing samples. We identified a set of transcripts that were exceptionally highly weighted on the first component. Overlaying these transcripts on RPF fold-change plots revealed that these transcripts largely corresponded, in each species examined, to a cluster of highly-expressed and strongly up-regulated genes independently identified (by visual inspection) as a group of genes of interest (**Fig. 5**C, Fig. S5). We investigated the identities of the transcripts making up this group and found that the significant majority corresponded to ribosomal protein genes, along with several core translation factors and a small number of additional genes including ubiquitin, heat shock proteins, and the RACK1 homolog (Table S2). These transcripts also formed a readily-identifiable cluster in mRNA-seq data, but with substantially weaker up-regulation (**Fig. 5**C, Fig. S5). Direct comparison of fold-changes for ribosomal proteins in mRNA and RPF data showed that up-regulation was significantly stronger at the RPF level, with average fold-changes >10 compared to ∼2-fold up-regulation at the mRNA level, indicating that the differential representation of these genes in the translatome was primarily due to translational regulation (**Fig. 5**D). We also found that non-ribosomal components of the core translation apparatus showed a significant trend towards up-regulation (**Fig. 5**C, red), with contributions from both increased mRNA abundance and translation efficiency (**Fig. 5**E).

Genes of the translation apparatus include many of the most highly-expressed transcripts. The coordinated up-regulation of these transcripts thus constitutes an extraordinary re-allocation of cellular energetic resources. From the raw counts for mapped RPF reads, we infer that, in *C. elegans*, approximately 3% of ribosomes are bound to a ribosomal protein transcript during L1 diapause. Three hours after feeding, more than 20% of all ribosomes are engaged in translating ribosomal proteins (Table S3). For the translation apparatus as a whole, this figure jumps from ∼4.5% in L1 diapause to nearly 30% in fed animals (Table S3). This striking change suggests that a central feature of the gene regulatory response to L1 diapause exit is to prioritize existing translational resources to building up the animal's capacity for protein synthesis.

In addition to translation genes, several categories of functionally related genes were enriched among transcripts whose RPF levels were significantly higher in developing animals. These include genes involved in promoting growth, development, ribosome biogenesis, the proteasome, and mitochondrial genes (Table S4). These enrichments were exceptionally consistent between the four species examined (Fig. S6A). In contrast, functional enrichments among genes with higher RPF levels in the L1 diapause state were generally weaker and less-consistent between species than those seen for transcripts up-regulated after feeding (Table S4, Fig. S6B). Manual inspection revealed a number of interesting genes that showed conserved higher expression during diapause, including *nhr-49* (required for adult reproductive diapause [3]–[6]), genes involved in autophagy and dauer formation, superoxide dismutases, and several heat-shock proteins (Table S5).

**Figure 6:**
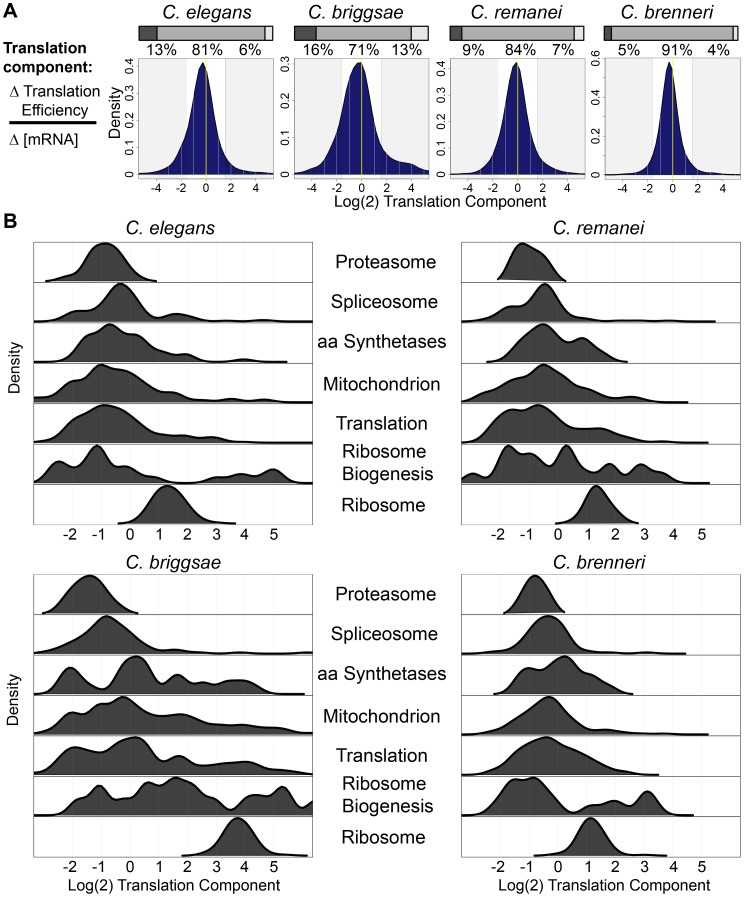
Functionally related gene sets are regulated by similar contributions of translational control and mRNA abundance changes in different species. (A) The distribution of the translation component of regulation for transcripts differentially-expressed in RPF data (fdr = 0.1) in the four species. Shaded regions correspond to changes that are >75% translational (right) and <25% translational (left). Pairwise comparisons showed statistically significant differences in the distributions between each species pair (p<2.2e-16; Kolmogorov-Smirnov test). The percentage of transcripts falling in these two regions and in between are shown above. Stacked barplots represent the fractions of transcripts in each shaded category. (B) Density distributions of the translational component of regulation for genes in several functional categories in the four species.

Ribosomal protein genes were subject to qualitatively similar regulation in each of the four species examined, i.e., modest increases in mRNA abundance and strong increases in translation efficiency. This led us to ask whether other groups of functionally related genes tended to share regulatory patterns across species. To this end, we defined the “translational component” of regulation as the ratio of the change in translation efficiency to the change in mRNA abundance (see Materials and Methods and Text S1). Examining the distribution of log(2)-transformed translational component scores for differentially-expressed transcripts in the four species revealed unimodal distributions in which a majority of transcripts (71–91%) were subject to “mixed” regulation, with mRNA abundance and translational regulation each accounting for at least 25% of the change in RPF level (**Fig. 6**A). Despite the overall similarities between the species, species-specific differences were evident. Notably, the broader distribution evidenced in *C. briggsae* indicated a greater tendency for transcripts to be primarily regulated either translationally or at the level of mRNA abundance, while the narrow distribution of *C. brenneri* suggested a trend toward coordinated regulation (**Fig. 6**A). While the results indicate a potential quantitative difference in the balance between transcriptional and translational regulation in different species, the coordination between these two regulatory modalities is evident in all four species.

We examined the distribution of translational components for transcripts corresponding to up-regulated functional gene categories and found that these categories showed remarkably similar profiles across the four species (**Fig. 6**B). For example, transcripts corresponding to proteasome components showed minimal contributions from translational regulation in each species, whereas ribosomal components, as demonstrated previously, exhibited large translational components (**Fig. 6**B). Likewise, spliceosome components favored strong contributions from mRNA accumulation changes in every species, while transcripts of the non-ribosomal translation machinery showed a broad distribution, indicating varied contributions from mRNA abundance and translation for this category. The between-species similarity of the relative contributions of mRNA abundance and translational control to regulation of functionally related transcripts suggests that the transcriptional and translational control networks underlying these changes may also be conserved in these species.

Our results point to a key role for translational control in the transition from L1 diapause to active development in these *Caenorhabditis* species. Translational regulation affects the expression of thousands of transcripts, and the patterns of regulation are well conserved between species at the genome-wide, functional, and gene level. Highly conserved translational modulation of certain sets of related transcripts, notably the ribosomal protein genes, implies that translational control programs may remain largely intact despite significant genome sequence divergence. A recent study reported the persistence of a pool of translationally repressed ribosomal protein mRNAs in yeast undergoing glucose starvation [13], suggesting that translational suppression of this class of genes may be a somewhat generalized feature of eukaryotic stress responses. In summary, we describe a system in which evolutionarily diverged species maintain a common program of mRNA abundance and translational efficiency changes that cooperatively drive the dynamic reallocation of gene expression resources to traverse a shared developmental and environmental transition.

## Materials and Methods

Strains were obtained from the Caenorhabditis Genetics Center: *C. elegans* N2 bristol, *C. briggsae* AF16, *C. remanei* PB4641, *C. brenneri* PB2801. Embryos were hatched in sterile S-complete liquid media, starved for 24 hours, and half were supplied with *E. coli* HB101. Samples were frozen in liquid nitrogen after 24 hours of starvation and after three hours of feeding. Three full biological replicates were prepared for each species. mRNA-seq and ribosome profiling were carried out as described in [23], [24], with modifications as described in Text S1. Sequencing was performed on Illumina's HiSeq 2000 machine.

Raw sequence reads were trimmed of adaptor sequence and mapped using Bowtie 0.12.7 [25] to the appropriate species' genomes and coding sequence with genomic flanking sequence, and screened for quality. For between-species comparisons, count normalization was performed with the EdgeR package [26] and orthologs were assigned using inParanoid [27]. Differential expression was determined using the DESeq package [28]. All additional analysis was carried out with custom Perl scripts and using the R computing environment [29]. Figures were created with R [30], [31].

Expression divergence for four-way orthologs was calculated by first normalizing log fold changes for each species by mean and standard deviation, then calculating each pair-wise species-species difference and taking the mean of the resulting differences. Principal components analysis was carried out using the prcomp function in R. Ontology analysis was performed using the web-based DAVID knowledge tool [32].

A more extensive description of the methods can be found in Text S1.

## Supporting Information

Figure S1 Transcriptome and translatome remodeling in *C. briggsae*, *C. remanei*, and *C. brenneri*. Expression mean versus log(2) fold change (MA) plots for mRNA and RPF abundance changes resulting from feeding-induced diapause exit. Colored points represent transcripts determined to be differentially expressed by a negative binomial test (fdr = 0.1) [28].
(EPS)

Figure S2 Pair-wise correlation of mRNA and RPF expression changes. Comparison of mRNA (gold) and RPF (blue) fold changes for orthologous transcripts in five species pairs. r_p_ = Pearson's correlation coefficient, r_s_ = Spearman's rho.
(EPS)

Figure S3 Controls for divergence metric: random shuffling of ortholog pairings. Ortholog assignments for four-way ortholog groups were shuffled by random sampling with replacement within each species. Divergence was then calculated for mRNA and RPF data. Effective normalization should yield identical distributions for RPF and mRNA data; unequal distributions would indicate that measured “divergence” is actually capturing inherent features of the data, for instance the larger overall fold-changes observed for RPF data. (A) Empirical cumulative distribution functions are plotted for four random shuffles. p-values: Kolmogorov-Smirnov (KS) test. (B) Smoothed distribution of K-S-derived p-values for one thousand random shuffles. Data smoothed by kernel density estimation, this is the reason for the presence of a small number of values >1.
(EPS)

Figure S4 Comparison of L1 diapause and early developing translatomes to continuously-fed, developing L2 translatomes. (A) Principal components analysis identical to that depicted in **Fig. 5**B except also including RPF abundance data from three samples of *C. elegans* L2 animals that had been continuously fed for >30 hours [23]–[24]. (B) Box plot of RPF abundances for ribosomal proteins in *C. elegans* L1 diapause, L1 developing, and L2 samples.
(EPS)

Figure S5 Differential expression of ribosomal proteins and translation factors in *C. briggsae* and *C. brenneri*. MA plots of expression changes for mRNA and RPF data with ribosomal proteins highlighted in blue, translation factors in red, and PC1 (genes heavily weighted on first principal component from PCA) genes in yellow.
(EPS)

Figure S6 Consistency of GO enrichments for up- and down-regulated genes across species. Heat map of negative log fdr values for the four species for functional categories enriched among transcripts that are more abundant in the developing state (A, blue) and more abundant in the diapause state (B, red). White squares represent categories that were not enriched, and thus not reported. All analysis was performed using DAVID web server [32].
(EPS)

Figure S7 Computational controls for **Fig. 2**C. Observed larger magnitude for RPF changes could have resulted from greater variance in RPF data. To address this, we examined the variance between replicates of RPF and mRNA-seq datasets by examining the distributions of fold changes between replicates. The density distribution of mean pair-wise fold changes for abundance values between three replicates is shown for RPF (red) and mRNA-seq (black) data in the four species. We observe that between-replicate differences are similar for RPF and mRNA-seq data, and of substantially lower magnitude than would be required to produce the effect seen in **Fig. 2**C.
(EPS)

Table S1 Data and quality for sequencing runs.
(XLSX)

Table S2 Table of genes with high weights on the first and second principal components of PCA of RPF data from four species.
(XLSX)

Table S3 Percentage of ribosome footprints mapping to ribosomal protein and translation genes in *C. elegans*.
(XLSX)

Table S4 Functional enrichments among genes with differentially-expressed RPF levels after diapause exit.
(XLSX)

Table S5 Manually-curated table of conserved, differentially-regulated genes of interest.
(XLSX)

Table S6 Strongly discordant *C. elegans* genes.
(XLSX)

Table S7 Table of Pearson correlation coefficients for mean fold changes of orthologs for mRNA-seq and RPF data.
(XLSX)

Text S1 Supplementary materials and methods and discussion.
(DOCX)
