# Supplementary material for: Conserved Translatome Remodeling in Nematode Species Executing a Shared Developmental Transition
Source: PLoS Genet. 2013 Oct 3;9(10):e1003739. doi: 10.1371/journal.pgen.1003739 (PMC3789828; doi:10.1371/journal.pgen.1003739)
Supplement: Text S1 — Supplementary materials and methods and discussion. (DOCX) [file pgen.1003739.s015.docx]

Supplementary Materials and Methods

I. Culturing and sample preparation

The following strains were obtained from the Caenorhabditis Genetics Center: *C. elegans* N2 bristol, *C. briggsae* AF16 , *C. remanei* PB4641, *C. brenneri* PB2801 . All strains were maintained on solid media as described in Brenner [34]. To generate a sufficient number of animals for starvation experiments, animals were grown in liquid culture in S-complete media [3] supplemented with *E. coli* HB101 at 20 C shaking at 180 rpm. Embryos were obtained by standard bleaching protocol, allowed to hatch for 24 hours in S-complete, and then fed for 72 hours until reaching adulthood. Hermaphroditic species were bleached at this point; gonochoristic species were transferred to enriched NGM solid media seeded with *E. coli* OP50 at 20 C for twelve hours to mate. Successive generations of liquid culture were grown until 36 million embryos were obtained.

Starvation experiments

36 million total embryos were added to sterile S-complete media at 20 C rotating at 180 rpm at a density of 20 embryos/μL and allowed to hatch for 24 hours. 18 million animals (arrested L1s) were harvested after 24 hours of starvation; remaining animals were fed with *E. coli* HB101 at 15 mg/mL for three hours before harvesting. Harvesting was accomplished by pelleting L1s by centrifuging at 950g for two minutes, washing several times with M9, and washing a final time in 300 mM NaCl. ~200 mg pellets of closely packed worms were flash frozen in liquid nitrogen and stored at -80C.

II. Molecular biology

mRNA-seq

Poly-A(+) RNA was prepared from frozen worm pellets using the Ambion micro-poly(A) purist kit frozen tissue protocol and sheared by alkali treatment with heat. RNA fragments of ~30 nt were cloned using the circligase protocol [10] with modification described below.

Ribosome profiling

Ribosome profiling and RNA extraction were performed as described in [23].

Circligase library preparation

Library preparation was performed as described in [23] with the following modifications:

1. In place of poly-adenylation, adaptor was ligated to 3' ends using T4 RNA ligase. 5.5 μL input RNA was denatured at 98 C for 45 seconds, placed on ice, mixed with 2 μL 5x adenylate ligase buffer [35], 1 μL DMSO, 0.5 μL 100 uM adenylated adaptor [10,35], and 1 μL μL T4 RNA ligase 1. Reaction was placed at 37 C for 45 minutes, heated to 98 C for 45 seconds, cooled on ice for >1 minute, supplemented with an additional 1 μL of ligase and incubated at 37 C a further 45 minutes.
2. Two rounds of circularization with 1 μL of circligase were carried out, each for 60 minutes at 60 C.
3. DNA oligos for circligase capture, for multiplexed Illumina Hi-seq:

3' linker: /5rApp/AGATCGGAAGAGCACACGTCT/3ddC/

PCR1: AATGATACGGCGACCACCGAGATCTACACTCTTTCCCTACACGACGCTCTTCCGATCT

PCR2: CAAGCAGAAGACGGCATACG

Reverse transcription: /5Phos/AGATCGGAAGAGCGTCGTGT/iSp18/CACTCA/iSp18/CAAGCAGAAGACGGCATACGAGATINDEX_RCGTGACTGGAGTTCAGACGTGTGCTCTTCCGATCT

The reverse complement of the barcode is inserted at the position indicated by brackets. We used indexes CGTGAT, ACATCG, GCCTAA, TGGTCA, CACTGT, ATTGGC, GATCTG, TCAAGT.

1. Sequencing was performed on Illumina HiSeq 2000 with multiplexing.

III. Informatics and analysis

Sequence reads were de-multiplexed using Illumina software and trimmed of 3' adaptor sequence by custom Perl scripts. Reference sequences for all species were version WS230 downloaded from Wormbase on February 20, 2012. Reads were mapped to genomes and collections of coding sequence plus 18 nt of flanking genomic sequence using Bowtie v0.12.7 [25] keeping only unique genomic alignments with one or fewer mismatches (parameters –v 1 –m 1) for genomic mapping and keeping all alignments with one or fewer mismatches (--strata and –v 1) for coding sequence alignments. Raw counts for each coding sequence were determined by first removing reads with multiple genomic mappings and then summing the number of reads aligning to each coding sequence.

All libraries were screened for quality based on the following requirements:

1) Must show three-nucleotide periodicity of 5' nt (ribosome profiling libraries only).

2) Must map overwhelmingly to coding sequence (ribosome profiling) or mRNA exons (mRNA-seq).

3) Visual inspection must reveal no evidence of strong amplification bottlenecking.

4) Gene counts must correlate well with replicate datasets.

Raw counts were normalized using the EdgeR package [26] and the “TMM” normalization method. Normalization was carried out independently for each species and experiment type (mRNA-seq or ribosome profiling). All starved and fed samples were members of experimental pairs, i.e., starved and 3h fed animals were collected from a single batch of worms, with half treated with food. To preserve this paired information, we computed fold changes separately for each biological replicate and used the mean of the fold changes (instead of the fold change of the means) where means are indicated. We note that for well-expressed transcripts these two methods of calculating mean fold change correlated extremely well.

Orthologs were assigned using data from InParanoid [27]. Tables of ortholog groups for all pair-wise species comparisons were downloaded from <http://inparanoid.sbc.su.se/>. The top protein pair was chose for each ortholog group with multiple paralogs to produce a table of one-to-one orthologs. From these tables, multispecies ortholog groups were constructed using the rules that 1) each member must be a 1:1 ortholog with at least one member of the group, and 2) no member can have a 1:1 ortholog in any species that is not a member of the group.

For differential expression analysis, we used DESeq’s implementation of count normalization and negative binomial test with multiple testing correction [28] separately on each experiment type and species. Ontology analysis was carried out using the web server of the DAVID bioinformatics resource (<http://david.abcc.ncifcrf.gov/>).

To calculate divergence of expression changes we began with the mean fold changes for each species for transcripts contained within four-way ortholog groups. Fold changes for each species were then normalized by the mean and standard deviation. Divergence for two species was taken as the absolute value of the simple difference in the normalized fold-change values between the species. For each ortholog group, divergence was calculated for all six pairwise species comparisons and the mean was taken as the divergence for the group. Shuffling ortholog assignments produced identical divergence distributions for mRNA-seq and RPF data (Fig. S3), indicating that this normalization procedure removes systematic differences in the fold-changes observed in the two types of data.

Supplementary text

We compared ribosome profiling data for diapause and early developing L1 animals to equivalent data from *C. elegans* having undergone >30 hours of continuous, fed development (L2 stage) [23,24]. First, we included data from all species as well as L2 *C. elegans* data in a second PCA, and found that the L2 samples had loadings on the first two components that were almost precisely intermediate to diapause and early developing L1s (Figure S4A). We further observed that RPF levels for ribosomal proteins in L2 samples were similarly intermediate to diapause and three hour post-feeding *C. elegans* samples (Fig. S4B). These results indicate that, taking the continuously-developing samples as the “normal” state, the translatomes of animals undergoing diapause and three hours post-diapause exit are both substantially altered. With respect to the ribosomal protein genes, these genes appear to be both potently suppressed relative to “normal” during diapause and also activated during recovery.
